# Enhanced enantioselectivity in halogen-bonding catalysis

**DOI:** 10.1038/s42004-023-00940-3

**Published:** 2023-07-04

**Authors:** Victoria Richards

**Affiliations:** Communications Chemistry, https://www.nature.com/commschem/

**Keywords:** Asymmetric catalysis, Homogeneous catalysis, Halogen bonding

## Abstract

Chiral halogen-bonding catalysts have emerged as a new approach towards asymmetric catalysis, but enantioselectivities have thus far remained low. Now, fine-tuning the substrate–catalyst halogen–halogen interactions is shown to significantly enhance enantioselectivity for a model anion-binding-catalyzed dearomatization reaction.

Chiral halogen-bonding catalysts offer intriguing opportunities in asymmetric catalysis, but the linear nature of the halogen bond creates a distance between the chiral catalyst and the substrate, hindering chirality transfer and leading to low enantioselectivities. Now, a team led by Olga García Mancheño at the University of Münster, Germany, show that fixing the substrate more strongly to the catalyst through an increased number of halogen–halogen bonding interactions enables more efficient chirality transfer and synthetically useful enantioselectivities (10.1002/anie.202304781)^1^.

The group previously reported a chiral tetrakis iodo-triazole system for enantioselective anion-binding catalysis^2^, whereby only one of the two catalyst arms was bound to the chloride ion in the catalyst–substrate ion-pair complex (Fig. 1a). They surmised that modifying their system to enable binding of the catalyst’s second arm would allow formation of a tighter complex and hence more efficient chirality transfer (Fig. 1b). Indeed, using a Reissert-type anion-binding catalyzed dearomatization as a model reaction, the team witnessed an impressive increase in enantioselectivity from 30% ee to up to 90% ee for their newly tailored system. “By introducing halogen substituents to the N-arene substrates, the formed halogen-arenium chloride salt is able to provide additional halogen–halogen interactions with the iodotriazoles of the catalyst through the originated σ-hole (opposite to the arenium rest) and its electron cloud (Lewis-base site)”, explains García Mancheño.

**Fig. 1:**
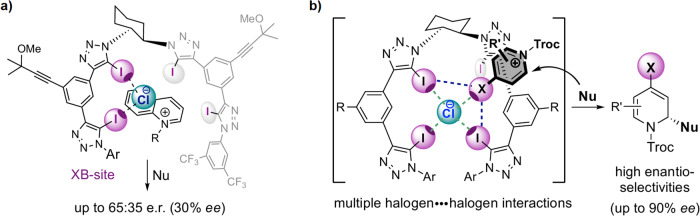
Substrate–catalyst halogen–halogen interactions. **a** Tetrakis iodo-triazole system where only one catalyst arm binds to the chloride ion in the catalyst–substrate ion-pair complex. **b** Tetrakis iodo-triazole system where both catalyst arms bind to the chloride ion in the catalyst–substrate ion-pair complex. Adapted with permission from Angew. Chem. Int. Ed. 10.1002/anie.202304781. Copyright (2023) Wiley.

“This application shows that halogen- and sigma-bonding activation could reach synthetically useful levels and become a powerful tool in asymmetric catalysis, which would lead to the activation of challenging substrates (ionic or neutral) and hence be interesting for a broad variety of synthetic applications”, says García Mancheño. The team additionally hope to use similar strategies to achieve high enantioselectivities in chalcogen-bonding catalysis.
